# Rhodanine-3-acetamide derivatives as aldose and aldehyde reductase inhibitors to treat diabetic complications: synthesis, biological evaluation, molecular docking and simulation studies

**DOI:** 10.1186/s13065-021-00756-z

**Published:** 2021-04-27

**Authors:** Mohsinul Mulk Bacha, Humaira Nadeem, Sumera Zaib, Sadia Sarwar, Aqeel Imran, Shafiq Ur Rahman, Hafiz Saqib Ali, Muazzam Arif, Jamshed Iqbal

**Affiliations:** 1 Department of Pharmaceutical Chemistry, RIPHAH Institute of Pharmaceutical Sciences G-7/4, Islamabad, Pakistan; 2 Department of Biochemistry, Faculty of Life Sciences, University of Central Punjab, Lahore, 54590 Pakistan; 3 Department of Pharmacognosy, RIPHAH Institute of Pharmaceutical Sciences G-7/4, Islamabad, Pakistan; 4 Centre for Advanced Drug Research, COMSATS University Islamabad, Abbottabad Campus, Abbottabad, 22060 Pakistan; 5 Department of Chemistry & Manchester Institute of Biotechnology, The University of Manchester, 131 Princess Street, Manchester, M1 7DN UK

**Keywords:** Rhodanine-3-acetic acid, Acetamide derivatives, Aldehyde reductase, Aldose reductase inhibitors, Molecular docking

## Abstract

In diabetes, increased accumulation of sorbitol has been associated with diabetic complications through polyol pathway. Aldose reductase (AR) is one of the key factors involved in reduction of glucose to sorbitol, thereby its inhibition is important for the management of diabetic complications. In the present study, a series of seven 4-oxo-2-thioxo-1,3-thiazolidin-3-yl acetamide derivatives **3**(**a–g**) were synthesized by the reaction of 5-(4-hydroxy-3-methoxybenzylidene)-4-oxo-2-thioxo-1,3-thiazolidin-3-yl acetic acid (**2a**) and 5-(4-methoxybenzylidene)-4-oxo-2-thioxo-1,3-thiazolidin-3-yl acetic acid (**2b**) with different amines. The synthesized compounds **3**(**a–g**) were investigated for their in vitro aldehyde reductase (ALR1) and aldose reductase (ALR2) enzyme inhibitory potential. Compound **3c**, **3d**, **3e**, and **3f** showed ALR1 inhibition at lower micromolar concentration whereas all the compounds were more active than the standard inhibitor valproic acid. Most of the compounds were active against ALR2 but compound **3a** and **3f** showed higher inhibition than the standard drug sulindac. Overall, the most potent compound against aldose reductase was **3f** with an inhibitory concentration of 0.12 ± 0.01 µM. In vitro results showed that vanillin derivatives exhibited better activity against both aldehyde reductase and aldose reductase. The molecular docking studies were carried out to investigate the binding affinities of synthesized derivatives with both ALR1 and ALR2. The binding site analysis of potent compounds revealed similar interactions as were found by cognate ligands within the active sites of enzymes.

**Supplementary Information:**

The online version contains supplementary material available at 10.1186/s13065-021-00756-z.

## Introduction

A number of long-term complications such as nephropathy, retinopathy, cataract and neuropathy have been associated with chronic diabetes. Nevertheless, damage to the blood vessels may be considered as one of the major long-term complications. Ergo, the patients with diabetes have somewhat a higher risk of developing cardiovascular diseases that might result in increased mortality because of microvascular complications such as strokes and peripheral artery disease [1]. Numerous pathways are involved in the impediments of diabetes mellitus, one of them being the glucolytic pathway. During normal physiological balance, this pathway is responsible for the regulation of metabolic flux of glucose concentration [2]. Whereas, polyol pathway, which is associated with the NADPH dependent reduction of glucose into sorbitol via aldol reductase, is known to be responsible for secondary complications of diabetes [3]. Therefore, in order to prevent the onset and to limit the progression of diabetic complications, aldol reductase (AR) has been considered as a drug target to develop AR inhibitor (ARIs).

**Fig. 1:**
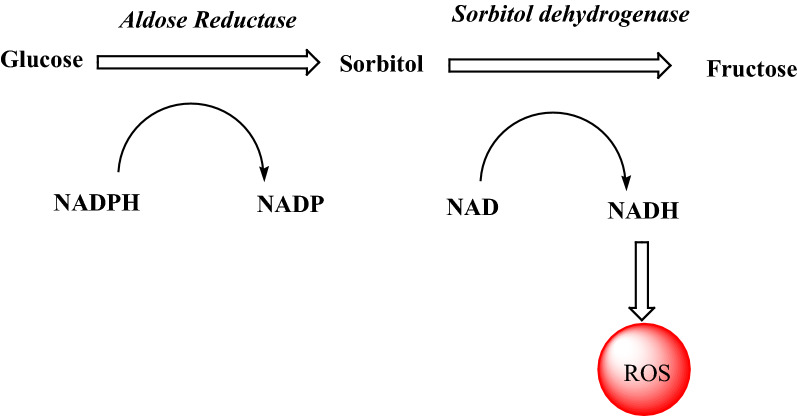
Role of aldose reductase (AR) in hyperglycemia-induced oxidative stress

Actually in polyol pathway, ALR1 (ALR1, EC 1.1.1.2) and, more importantly, its closely related homolog ALR2 (EC 1.1.1.21) convert glucose to sorbitol, which is then oxidized to fructose by sorbitol dehydrogenase (l-iditol: NAD + 5-oxidoreductase, EC 1.1.1.14, SD). Normally, under euglycemic conditions, a very non-significant conversion of glucose to sorbitol take place via this pathway as ALR2 shows a low substrate affinity for glucose. Preferably, glucose is phosphorylated using ATP by hexokinase of glycolytic pathway rather than ALR2 as the former possess greater substrate affinity than later. However, hexokinase becomes saturated quickly under hyperglycemic conditions consequently polyol pathway becomes operative (Fig. 1). Therefore, formation of sorbitol becomes rapid as compared to its conversion into fructose. The polarity of sorbitol hinders it from entry into the membranes which results in its removal from tissue by diffusion. Accumulation of sorbitol within the cell causes cell osmolarity to increase.

Increased polyol activity also results in significant imbalance in the cytosolic coenzyme rations; NADPH/NADP+ and NAD+/NADH. A state of pseudohypoxia is induced as a result of this alteration in the redox state of pyridine nucleotides that contribute towards the onset of hyperglycemic oxidative stress via accumulation of reactive oxygen species. Consequently, diabetic tissue injury and dysfunction occurs due to the activation of several downstream mechanisms such as mitogen-activated protein kinases (MAPKs), poly (ADP-ribose) polymerase (PARP) and protein kinase C (PKC) isoforms by these reactive oxygen species. Since the levels of fructose are also alleviated by polyol pathway, these complications are further accelerated as fructose and its metabolites are 10 times more potent non-enzymatic glycation agents than glucose [4–10]. In conclusion, the oxidative as well as the osmatic stress, generated by the activation of ALR2, contributes to the diabetic impairments, mainly affecting ocular, renal, vascular and nervous systems. It has been reported previously that most of the known ALR2 inhibitors (ARIs) also inhibit ALR1 demonstrating the fact that there are few common features in the active sites of both the enzymes by which the bind the inhibitor and substrate. A lot of ARIs have already been reported in the studies such as carboxylic acid derivatives and hydantoin with low IC_50_ values in micromolar and sub-micromolar ranges [11], yet the only known ALR2 inhibitor that is being marketed only in Japan for the treatment of diabetic neuropathy is epalrestat, a rhodanine-3-acetic acid derivative [12]. However, undesirable effects like hypersensitivity and Steven-Johnson syndrome are associated with these hydantoin type of inhibitors [13]. Recently Del-Corso A et al. reported the differential inhibition of AR using different molecules having both hydrophilic and lipophilic scaffolds present [14]. Furthermore, most of them consist of a chemical group of acetic acid on the core. However, a lower tissue penetration has been found to be the major shortcoming for some individual potent carboxylic acid ARIs [15, 16]. Therefore, it is proposed to check carboxylic acid derivatives including amides to be tested for AR inhibition. This approach can reduce the side effects associated with the use of AR inhibitors.

Rhodanine derivatives are known to possess various biological activities which include β-lactamase inhibitory potential [17], inhibitors of (JSP-1) JNK-stimulating phosphatase-1 [18], histidine decarboxylase inhibitors [19], anti-apoptotic action [20], antibacterial activity [21], fungicidal activity [22], HIV-1 integrase inhibitory activity and HIV-1 cell replication inhibition [23] and trypanocidal activity [15]. These compounds can stimulate the formation of parathyroid hormone, receptor-mediated cAMP and may be useful in the treatment of degenerative arthritis, osteoarthritis and rheumatoid arthritis both locally and systemically [16]. Previously, rhodanine-3-acetic acid derivatives have been screened biologically against various targets including aldose reductase which resulted epalrestat being used in Japan for diabetic complications. Still there is a need to develop new AR inhibitors with better efficacy and safety profile which can overcome the complications in diabetic patients.

Since rhodanine is an essential moiety for AR inhibition, the present study was designed to synthesize new 5-Benzylidene-(4-oxo-2-thioxo-1,3-thiazolidin-3-yl) acetamide derivatives and explore their potential against aldehyde/aldose reductase enzymes. In silico studies were performed to investigate the binding mode of synthesized compounds with target enzymes ALR1 and ALR2.

## Results and discussion

### Chemistry

**Scheme 1:**
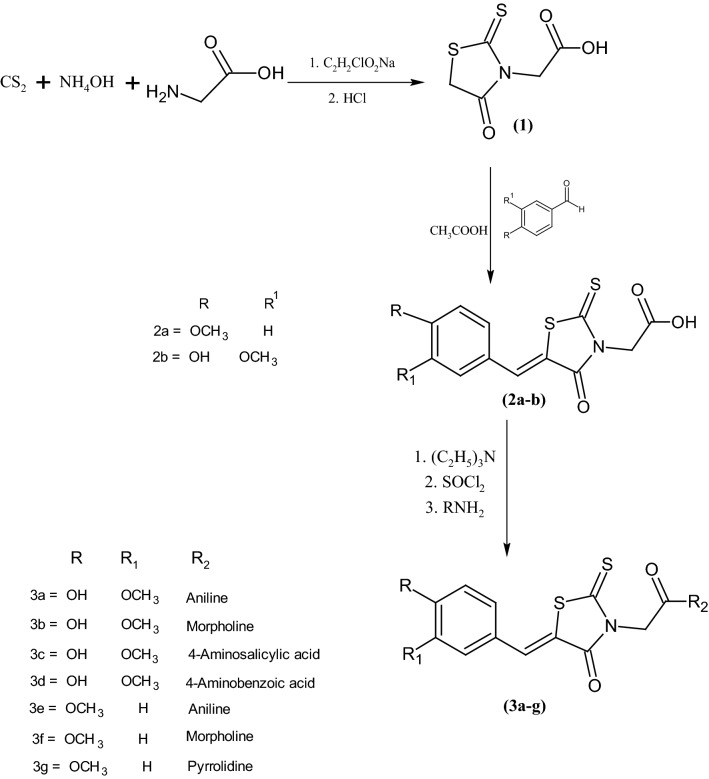
Synthesis of Rhodanine-3-acetamide derivatives

Synthesis of rhodanine-3-acetic acid (**1**) was accomplished in two steps by reported procedure. The resultant product was condensed with aromatic aldehydes (vanillin and 4-methoxy benzaldehyde) in the presence of few drops of glacial acetic acid to get the corresponding benzylidene derivatives **2**(**a–b**). These benzylidene derivatives were treated with thionyl chloride and finally with respective amines in the presence of triethylamine to furnish the target carboxamide derivatives **3**(**a–g**) (see Scheme 1). All the synthesized acetamide derivatives were characterized by FTIR and ^1^H NMR data. IR data showed amide cabonyl stretchings in the range 1610–1650 cm^−1^. C=S stretching vibrations were observed at 1200–1300 cm^−1^. In the ^1^H NMR spectra of these compounds, methylene protons of acetamide group resonated at 4.42–5.47 ppm and signals at 7.71–7.84 ppm were assigned to vinylic protons of C=CH confirming the formation of benzylidene derivatives. All the compounds exhibited singlet of methoxy protons above 3.4 ppm. Rest of the peaks were observed at expected position in the respective IR and NMR spectra.

### ALR1 and ALR2 enzyme inhibition

The newly synthesized of 5-benzylidene rhodanine-3-acetamide derivatives **3**(**a–g**) were evaluated for in vitro enzyme inhibitory potential on aldehyde and aldose reductase enzymes. Valproic acid for aldehyde reductase and sulindac for aldose reductase was used as reference drugs with sodium d-glucoronic acid and d,l-glyceraldehyde as substrate, respectively. IC_50_ ± SEM (µM) values were calculated for the compounds showing more than 50% inhibition against both the isozymes, (Table 1).

**Table 1:** Aldehyde and aldose reductase inhibition efficacy by 5-benzylidene rhodanine-3-acetamide derivatives **3**(**a–g**)

Codes	R1	R2	R'	ALR1	ALR2
				IC_50_ ± SEM (µM)/% inhibition	
**3a**	OH	OCH_3_	C_6_H_5_NH_2_	5.38 ± 0.07	0.25 ± 0.04
**3b**	OH	OCH_3_	C_4_H_9_NO	6.07 ± 0.05	19.2 ± 0.08
**3c**	OH	OCH_3_	C_7_H_7_NO_3_	2.38 ± 0.02	6.38 ± 0.01
**3d**	OH	OCH_3_	C_7_H_7_NO_2_	3.76 ± 0.09	7.36 ± 0.02
**3e**	OCH_3_	H	C_6_H_5_NH_2_	2.87 ± 0.01	12.71 ± 0.09
**3f**	OCH_3_	H	C_4_H_9_NO	2.18 ± 0.03	0.12 ± 0.01
**3g**	OH	OCH_3_	C_4_H_9_N	2.22 ± 0.04	2.42 ± 0.03
Valproic acid				57.4 ± 0.89	–
Sulindac				–	0.29 ± 0.08

All the synthesized compounds **3a–g** exhibited good inhibitory activity against both aldehyde reductase and aldose reductase. Especially the IC_50_ values of all derivatives are lower than the standard valproic acid for ALR1. Compounds **3c** and **3f** with an IC_50_ value of 2.38 ± 0.02 µM and 2.18 ± 0.03 µM, respectively showed the significant inhibition. Similarly, compounds **3a**, **3b** and **3e** have shown good inhibition towards aldehyde reductase. As far as ALR2 is concerned all the compounds exhibited moderate activity especially **3a** and **3f** were more active than the standard sulindac with IC_50_ of 0.25 ± 0.04 and 0.12 ± 0.03 µM, respectively. If we look at the selectivity of our synthesized derivatives towards ALR1 and ALR2, it was observed that compound **3a** and **3f** are selective towards ALR2 while **3c**,** 3d** and **3e** are more selective and potent inhibitors of ALR1.

**Fig. 2:**
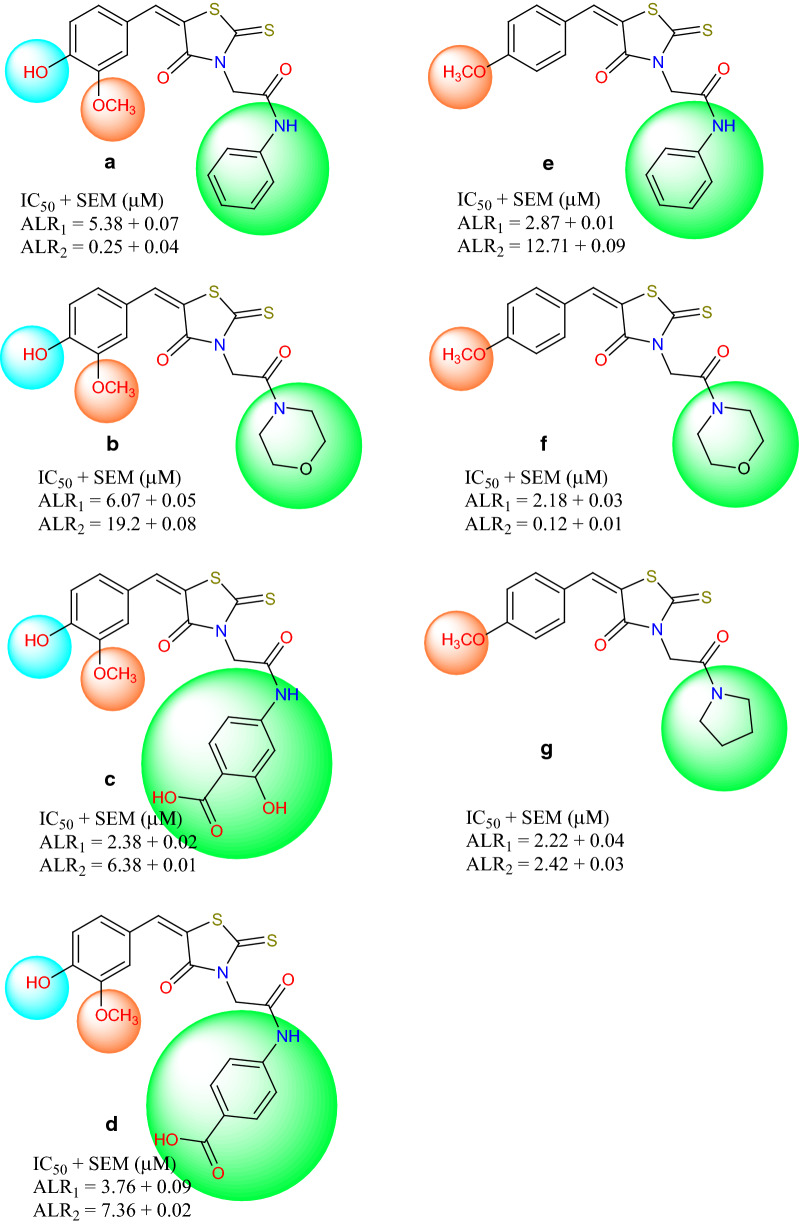
Structures with potency of synthesized derivatives **3**(**a–g**)

Furthermore, it was observed that 4-methoxybenzylidene derivatives are less selective between two isozymes while other derivatives bearing vanillin moiety **3**(**a–d**) showed more selective behavior towards ALR1 and ALR2. While comparing **3a** with **3b** it suggests that the phenyl group is beneficial for ALR2 activity in the presence of additional methoxy and hydroxyl group, however the opposite seems to be the case for **3e** and **3f** as in these cases, additional substituent is only methoxy group. If we look at the amide substitution of all compounds, it is evident that carboxylic group containing acetamides **3c** and **3d** are more active against ALR1 while the presence of morpholine and pyrrolidine moiety increases the inhibitory potential of compounds against both isozymes with lesser selectivity. The pictorial representation of inhibition profile and the influence of various groups attached to synthetic compounds is presented in Fig.2

### Docking analysis

**Fig. 3:**
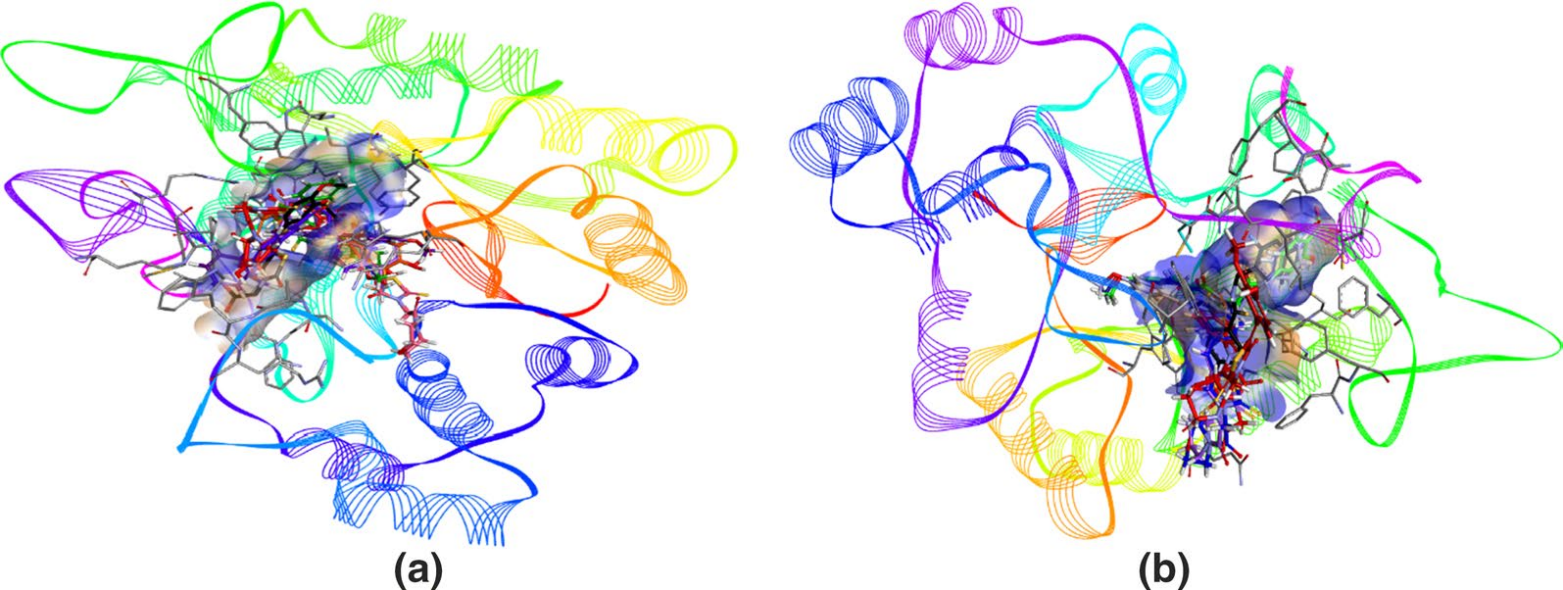
The overlap of all the docked inhibitors **3**(**a**–**g**) inside the active site of aldehyde reductase (ALR1) (**a**) having FX4401 (black), **3a** (green), **3b** (pink), **3c** (orange), **3d** (blue), **3e** (indigo), **3f** (red) and **3g** (light purple) in the presence of NAP350 (beige) and aldose reductase (ALR2) (**b**) having IDD594 (black), **3a** (blue), **3b** (light blue), **3c** (light green), **3d** (brown), **3e** (grey), **3f** (red) and **3g** (light purple) in the presence of NADP + (beige) with aromatic surface representation

For docking analysis, the protein structures were selected from protein databank. In case of aldose reductase, the crystal structure of human ALR2 was available (1US0) and downloaded. However, human aldehyde reductase crystal structure was not available and porcine aldehyde reductase structure (3FX4) was selected (as human and porcine show about 97% sequence homology). For the purpose of validation, before carrying out the docking studies, the cognate ligands of both the enzymes were extracted and docked inside the active site. After docking, root mean square deviation of co-crystallized ligands (FX4401 for 3FX4 and IDD594 for 1US0) was found less than 1.0 Å for the respective enzymes. After reproducing the cognate ligands and their binding poses inside the active pocket, the docking studies were performed. The in vitro results clarified that **3e** was potent and selective inhibitor of ALR1, whereas, **3a** was selective as well as potent inhibitor of ALR2. However, some dual inhibitors were found, and docking analysis was carried out against those dual inhibitors, **3f** and **3g** in the active site of both the receptors. After evaluation of all the docked poses by visual inspection, the poses were selected based on interactions shown inside the active pockets of aldose reductase and aldehyde reductase. The 3D interaction poses suggested that compounds presented good affinity for the target enzymes. Figure 3 depicts the overlap of all the selected compounds docked inside the active site of ALR1 and ALR2, respectively.

**Fig. 4.**
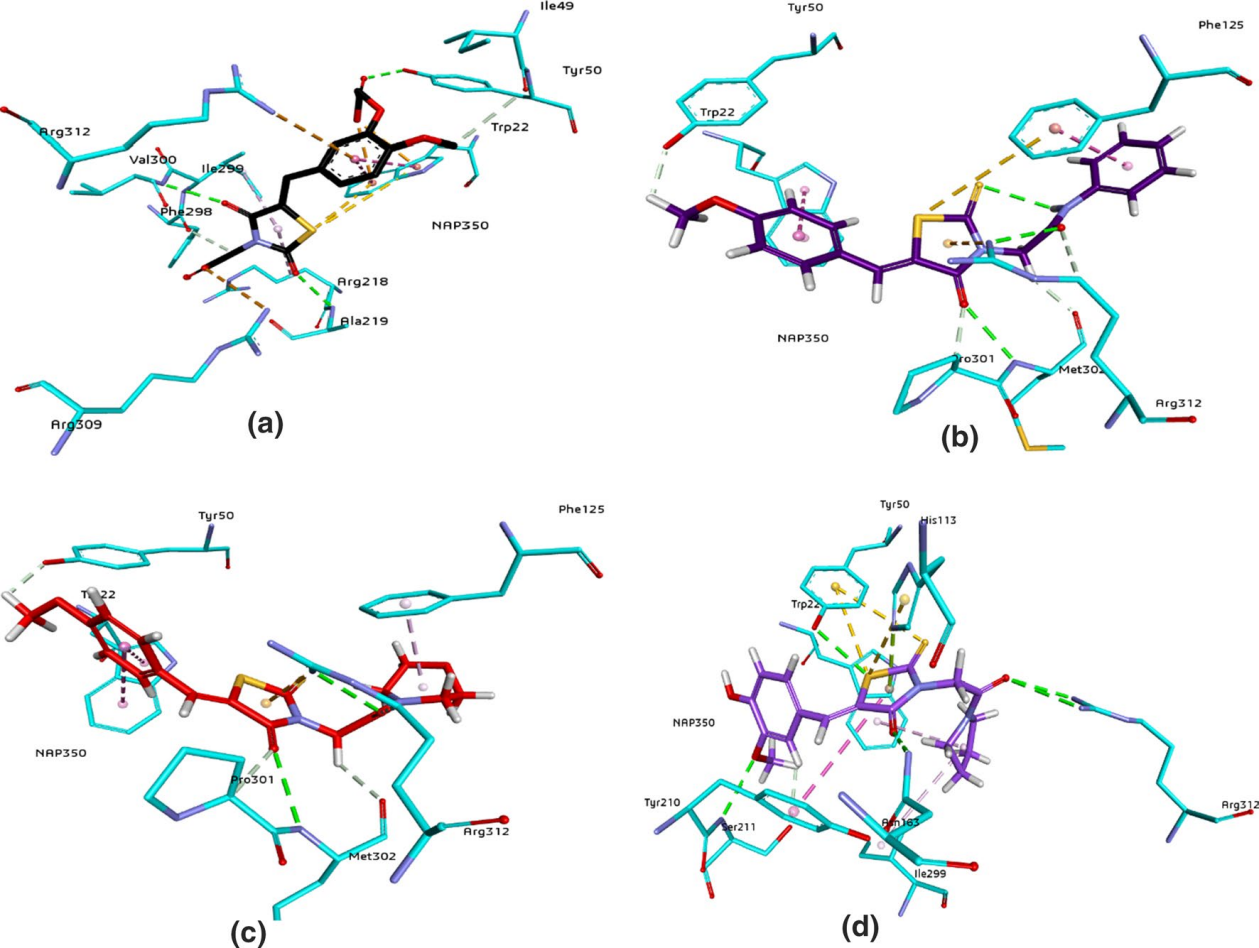
3D interactions of cognate ligand (**FX4401**) (**a**), selective inhibitor (**3e**) of ALR1 (**b**) and dual inhibitors (**c**: **3f**; **d**; **3g**) inside the active pocket of 3FX4. The interactions are represented by green (conventional hydrogen bonding), yellow (pi–sulfur interactions), tea pink (pi–pi T shaped and pi–pi stacked interactions), and grey (carbon–hydrogen bonding)

**Fig. 5.**
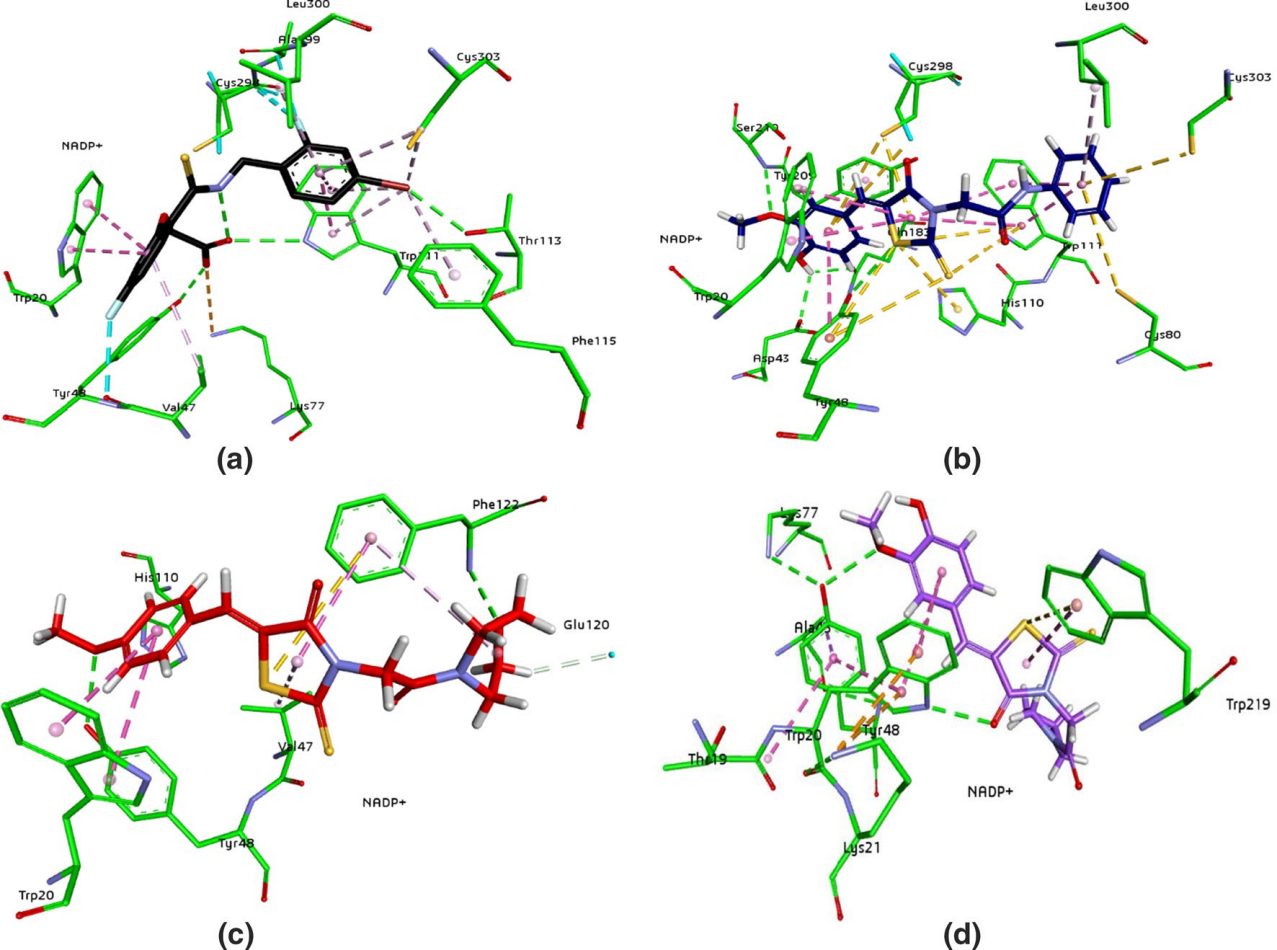
3D interactions of cognate ligand (IDD594) (**a**), selective inhibitor (**3a**) of ALR2 (**b**) and dual inhibitors (**c**: **3f**; **d**; **3g**) inside the active pocket of 1US0. The interactions are represented by green (conventional hydrogen bonding), yellow (pi–sulfur interactions), tea pink (pi–pi T shaped and pi–pi stacked interactions), and grey (carbon–hydrogen bonding)

The active pockets were selected after analysis of binding interactions of co-crystallized ligands within the active site. The 3D interaction diagrams of co-crystallized ligand, selective inhibitor and dual inhibitors of ALR1 are shown in Fig. 4, while the 3D interaction diagrams of co-crystallized ligand, selective inhibitor and dual inhibitors of ALR2 are presented in Fig. 5. The results showed that all the docked inhibitors are involved in a network of hydrogen bonding interactions with the target enzymes. Compound **3a** has greater hydrogen bonding interactions against ALR2 while compound **3f** is forming more hydrogen bonds with ALR1.

The detailed analysis of ALR1 co-crystallized ligand and the inhibitors showed that Trp22 and Tyr50 were the most important residues showing interaction with the inhibitors within the active site. The cognate ligand presented the same interactions and our selective inhibitor, **3e** was making π–π interactions with Tyr50. The experimental results suggested that compound **3a** showed greater inhibitory potential against aldose reductase (ALR2) as compared to ALR1, while compound **3e** was more active against ALR1 as compared to ALR2. Inside the binding site of ALR1, the oxygen atom of 4-oxo-2-thioxo-1,3-thiazolidine ring of compound **3e** (Fig. 4) make two hydrogen bonds with amino acid Arg312 and Met302. Two π–π stacked bonds were formed by the aromatic part of rhodanine ring with Phe125 and Trp22. While amino acid Phe125 showed backbone donor interaction with the sulfur of rhodanine ring. The selected compounds showed the binding interactions necessary for the inhibition of ALR1 within the active site.

When ALR2 active site residues and interaction analysis of selected compounds were investigated, it was noted that the compounds exhibited the interactions that were reported in the literature [24, 25] and are responsible for the inhibitory behavior towards ALR2. In addition to Typ111 and Trp20, His110 and Tyr48 are also important to show interactions and are playing key role towards the inhibition of enzyme. Moreover, compound **3a** (Fig. 5) showed five hydrogen bonds and make stronger interaction with ALR2 binding pocket. Each H-bonds with Gln183, Asp43, Ser210 by the aromatic part of rhodanine ring, while Tyr48 forming H-bond with the sulfur of rhodanine ring and last one with carbonyl oxygen of rhodanine by Trp111. The aromatic ring makes a π-π interaction with Tyr209, and hydroxyl group makes hydrogen bond with Gln183. The cysteine residues in the active pocket of ALR2 and sulfur atoms of the compound **3a** are involved in forming multiple pi-sulfur interactions with Cys303, Cys80, Trp111, His110, Tyr48, respectively. These additional pi-sulfur interactions could be the reason of high potency. The formation of strong hydrogen bond strengthens the potent inhibitor inside the active pocket and contribute towards the potent inhibition towards isozyme. The docking scores of all the derivatives against ALR1 and ALR2 have been given in Table 2. 2D interaction diagrams are provided in the supporting information (see Additional file 1: Figure S1).

**Table 2:** Docking score of the top pose of all the synthesized compounds and reference drugs in the ALR1 & ALR2

Codes	ALR1	ALR2
	Docking score by FlexX for top pose (kcal mol^‒1^)	
**3a**	−26.0700	−31.5855
**3b**	−28.4363	−24.3437
**3c**	−28.7833	−25.3824
**3d**	−24.6395	−23.4514
**3e**	−33.9628	−25.9504
**3f**	−30.1254	−30.1640
**3g**	−29.7386	−30.3788
Valproic acid	−18.5923	–
Sulindac	−	−24.3431

### Molecular dynamic simulations

**Fig. 6:**
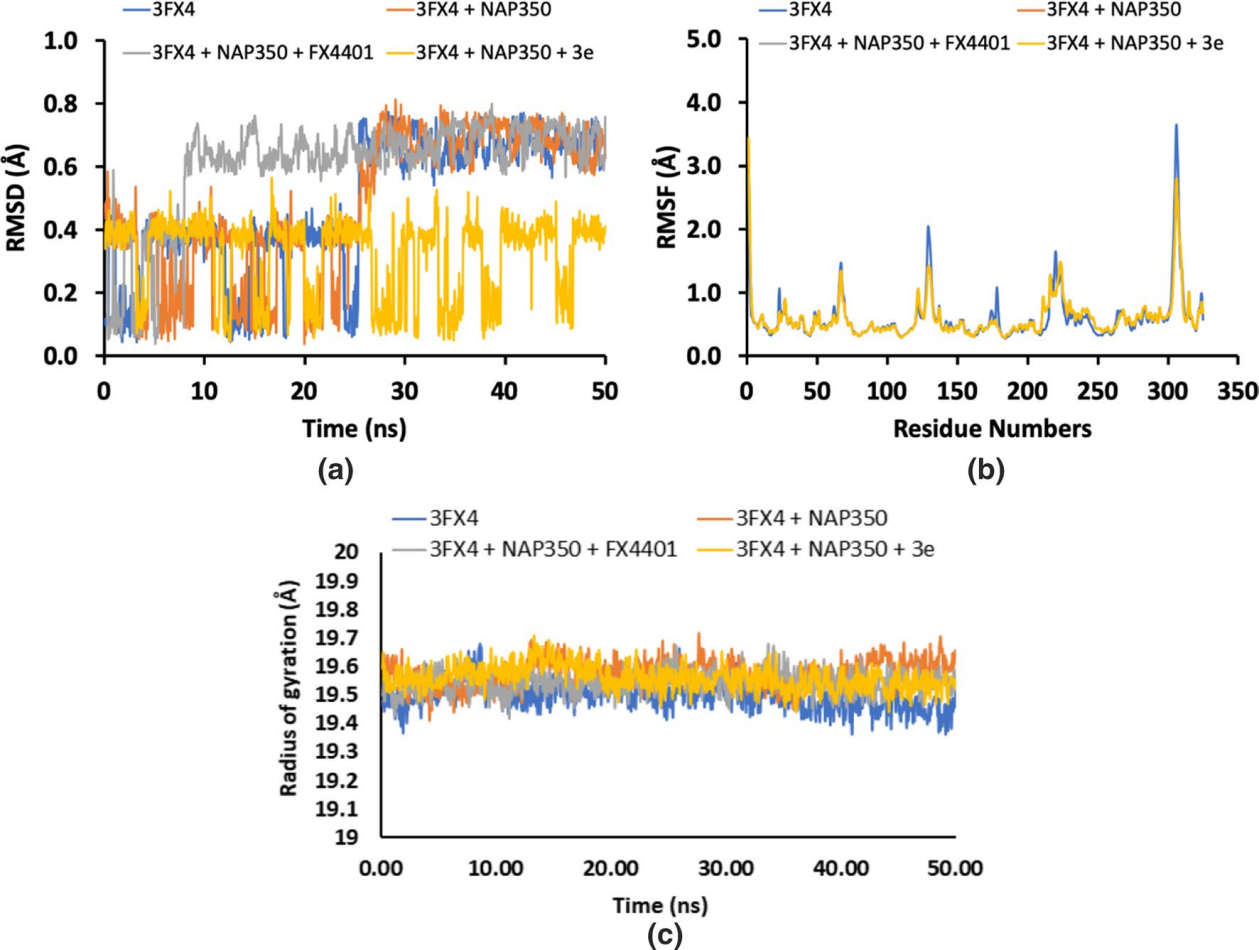
Root Mean Square Deviation (RMSD) (**a**), Root Mean Square Fluctuation (RMSF) (**b**) and radius of gyration (**c**) of amino acid residues of 3FX4, protein + cofactor, protein + cofactor + cognate ligand and protein + cofactor + selective inhibitor (**3e**) during 20 ns MD-simulation run

**Fig. 7:**
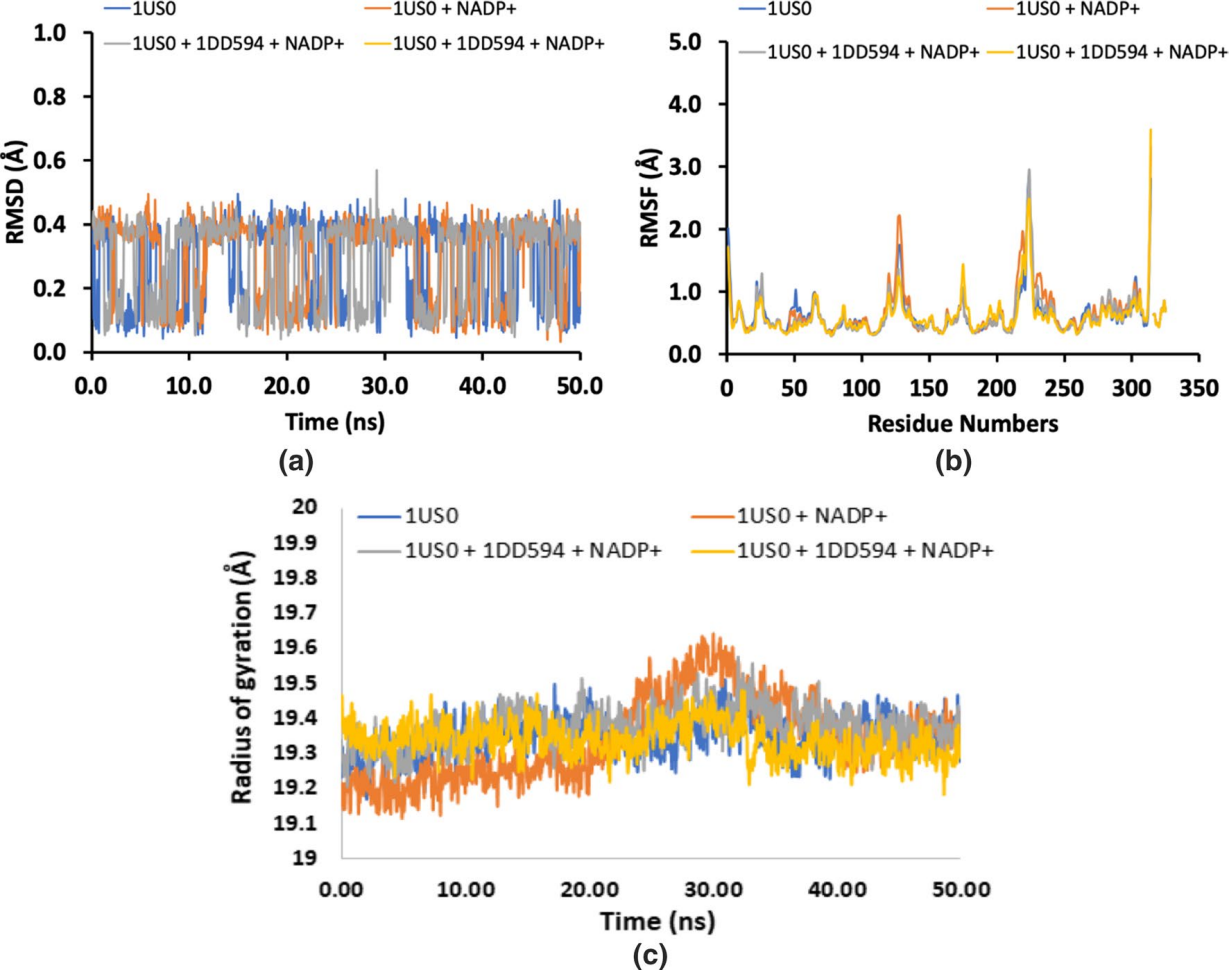
Root Mean Square Deviation (RMSD) (**a**), Root Mean Square Fluctuation (RMSF) (**b**) and radius of gyration (**c**) of amino acid residues of 1US0, protein + cofactor, protein + cofactor + cognate ligand and protein + cofactor + selective inhibitor (**3a**) during 20 ns MD-simulation run

In order to justify the docking analysis, the molecular dynamic simulations were performed for both the enzymes (ALR1 and ALR2). The structure of proteins (apo) were first subjected to MD run of 50 ns and then docked poses of selected ligands were submitted to MD run. The results in the form of RMDS values showed that the structural of protein and protein–cofactor complex attained stability after 25 ns MD simulation while the complex of protein with cofactor and cognate ligand attained stability after 10 ns MD simulation, which mean cognate ligand helps protein-cofactor complex to attained stability quickly. On the other hand the protein-cofactor complex with inhibitor shown fluctuations with low RMSD values throughout 50 ns MD simulation (Fig. 6). However, the RMSD plot of ALR2 showed that the trajectories of apo and holo forms (protein + cofactor) (protein + cofactor + cognate ligand) (protein + cofactor + selective inhibitor) showed less fluctuations after 4 ns and both the plots were found stable afterwards (Fig. 7). When root-mean-square fluctuation (RMSF) of ALR1 and ALR2 were examined, it was observed that residues were found stable in both the cases (Figs. 6 and 7). Moreover, the radius of gyration for both the proteins in their apo and holo forms were provided in Figs. 6 and 7. The results of docking studies revealed that the after binding of potent compounds inside ALR1 and ALR2, the tight complex formation made the complex stable and resulted in lowering of the energy. Our results suggested that docked complex throughout MD trajectories exhibit stable behavior and therefore, increasing the efficacy of docked poses and, hence docking results.

### ADME properties

ADME properties predict the impact of therapeutic compounds to access the target considering some parameters. These properties were evaluated using several prediction tools [26]. The properties help determining the druglikeness of compounds being used for drug discovery and development by sorting out new druggable candidates that are safer and follow the effective rules used for determination of these parameters. The properties suggested that our synthesized derivatives are safer to use as drug and have high probability of blood brain penetration and absorption (Table 3).

**Table 3:** ADME prediction scores for the synthesized compounds and reference drugs

Properties	Compounds								
	**3a**	**3b**	**3c**	**3d**	**3e**	**3f**	**3g**	Valproic acid	Sulandic
Mol. Wt. (g/mol)	400.47	394.47	460.48	444.48	384.47	378.47	378.47	144.21	356.41
No. of atoms	27	26	31	30	26	25	25	10	25
No. of aromatic atoms	12	6	12	12	12	6	6	0	12
No. of rotatable bonds	6	5	7	7	6	5	5	5	4
No. of H-bond acceptors	4	5	7	6	3	4	4	2	4
No. of H-bond donors	2	1	4	3	1	1	1	1	1
TPSA (Å^2^)	136.26	109.77	193.79	120.54	116.03	116.47	127.47	37.30	73.58
Log *P*_o/w_ (iLOGP)	3.09	3.14	2.32	2.29	3.18	3.11	3.10	1.99	2.75
GI absorption	High	High	Low	Low	High	High	High	High	High
BBB permeant	No	No	No	No	No	No	No	Yes	No
P-gp substrate	No	No	No	No	No	No	No	No	No
Lipinski	No violation	No violation	No violation	No violation	No violation	No violation	No violation	No violation	No violation
Veber	No violation	No violation	No violation	No violation	No violation	No violation	No violation	No violation	No violation
Bioavailability score	0.55	0.55	0.11	0.11	0.55	0.55	0.55	0.85	0.85
PAINS	No alert	No alert	No alert	No alert	No alert	No alert	No alert	No alert	No alert
Leadlikeness	No violation	No violation	No violation	No violation	No violation	No violation	No violation	No violation	No violation

## Conclusion

5-Benzylidenerhodanione-3-acetamide derivatives were successfully synthesized and characterized in this study. The synthetic analogues were screened against aldose reductase and aldehyde reductase and compound **3f** was found dual inhibitor exhibiting an IC_50_ values of 0.12 ± 0.01 and 2.18 ± 0.03 µM, respectively. However, compound **3a** with an IC_50_ value of 0.25 ± 0.04 µM was selective inhibitor of aldose reductase and compound **3e** with an IC_50_ value of 2.87 ± 0.01 µM was selective inhibitor of aldehyde reductase. All the newly synthesized rhodanine-3-acetic acid derivatives, including amide functionality, showed relatively low IC_50_ values which suggests low toxicity and less side effect, which can obviously be established only after further investigation. In silico analysis with human aldose reductase (PDB ID: 1US0) and aldehyde reductase (PDB ID: 3FX4) was carried out with these compounds which further supported the results of in vitro study. Overall, the computational studies of the synthesized compounds and in vitro enzyme inhibitory studies against ALR1 and ALR2 identified some potent compounds which can be used as lead molecules for further development to treat diabetic complications.

## Experimental

## Materials and methods

All the reagents were purchased from Sigma Aldrich and Alfa Aesar and used without further purification. Melting points of the synthesized compounds were recorded using Gallenkamp melting point apparatus. Characterization of the synthesized compounds was done by FTIR and ^1^H NMR and elemental analysis data. FTIR spectra were recorded on Thermoscientific NICOLET IS10 spectrophotometer, and ^1^H NMR spectra were taken on Bruker AM300 MHz spectrophotometer, in which DMSO was used as solvent. The progress of reaction was monitored by TLC with pre-coated silica gel 60 F254 plates using ethyl acetate and petroleum ether as mobile phase.

### General procedure for the synthesis of Rhodanine-3-acetic acid (1)

Glycine (2.4 g, 0.031 mol) was dissolved in 33% NH_4_OH (20 ml), carbon disulfide (2.36 g, 0.031 mol) was added to the solution and stirred vigorously for 1 h while color of the solution turned orange. Then aqueous solution of sodium chloroacetate (3.61 g, 0.031 mol) was added and refluxed for 3 h, after completion, reaction mixture was acidified with dilute HCl to bring the pH to 1.0 and further refluxed for 1 h. Saturated NaHCO_3_ solution was added to the reaction mixture to neutralize it and the resultant solution was acidified again with dilute HCl. The solid separated was filtered and recrystallized with water to obtain rhodanine-3-acetic acid. Yield: 86%. M.p.: 145–148 °C [27].

### General procedure for the synthesis of 5-benzylidenerhodanine-3-acetic acid 2(a–b)

Equimolar amounts of rhodanine-3-acetic acid, anhydrous sodium acetate and respective aldehyde were dissolved in glacial acetic acid (30 ml) and solution was put to reflux for 3–4 h. After completion the reaction mixture was cooled and the solid separated was filtered, washed with water and recrystallized from ethanol [28].

#### Z-5-(4-hydroxy-3-methoxybenzylidene) rhodanine-3-acetic acid (2a)

Yield: 75%. M.p.: 144 °C [28].

#### (Z)-5-(4-methoxybenzylidene) rhodanine-3-acetic acid (2b)

Yield: 66%. M.p.: 249–250 °C.

### General procedure for the synthesis of 5-benzylidenerhodanine-3-acetamide derivatives 3(a–g)

The synthesized 5-benzylidenerhodanine-3-acetic acid **2**(**a–b**) was stirred with excess of thionyl chloride in dichloromethane (20 ml) for 2 h. After reaction completion solvent was evaporated and residue treated with equimolar amount of respective amine in the presence of triethylamine and dichloromethane as solvent. Progress of reaction was monitored by TLC, after completion product was isolated by evaporation and purified by column chromatography [29]**.**

### (Z)-5-(4-hydroxy-3-methoxybenzylidene)-3-(2-anilino-2-oxoethyl)-2-thioxothiazolidin-4-one (3a)

Yield 53.5%, Orange solid, m.p 201–202 ℃, R_f_ 0.42 (ethyl acetate:pet-ether 2:1), IR (KBR) cm^−1^, 1635 (C=O), 1201 (C=S), 3440 (NH), 1020 (C–N), 3150 (OH), 1489 (CH_3_), ^1^H NMR (DMSO-d_6_, 400 MHzδ ppm) 9.98 (s, 1H, OH), 7.51 (s, 1H, vinylic H) 7.15–7.46 (m, 7H, ArH), 7.01 (d, 1H, J = 8.7 Hz, ArH), 6.87 (d, 2H, J = 8.7 Hz, Ar–H) 5.52 (s, 2H, CH_2_–CO) and 3.65 (s, 3H, OCH_3_), ^13^C NMR (DMSO-d_6_, 100 MHz, δ ppm): 149.7, 146.2, 134.3, 126.8, 116.7, 109.2, 138.4, 129.7, 129.7, 125.1, 119.9, 119.9 (Ar–C), 194.3, 167.2, 130.2 (thiazolidine-C), 162.6 (CONH), 133.7 (CH), 57.1 (OCH_3_), 48.8 (CH_2_), Anal. Calcd. For C_19_H_16_N_2_O_4_S_2_: C, 56.93; H, 4.02; N, 6.99; S, 15.98; Found: C, 56.84; H, 3.99; N, 7.00; S, 15.99.

### (Z)-5-(4-hydroxy-3-methoxybenzylidene)-3-(2-morpholino-2-oxoethyl)-2-thioxothiazolidin-4-one (3b)

Yield 61.5%, yellow solid, m.p 211–213 °C, R_f_ 0.41 (ethyl acetate:pet-ether 2:1), IR (KBR) cm^−1^, 1615 (C=O), 1300 (C=S), 1104 (C–N), 1192 (C–O), 3384 (OH), 1450 (CH_3_), ^1^H NMR (DMSO–d_6_, 400 MHzδ ppm) 9.76 (s, 1H, OH), 7.80 (s, 1H, vinylic H), 7.37–7.43 (m, 1 H, ArH), 7.14–7.22 (m, 1 H, ArH), 7.02 (d,d 1H, J = 8.4 Hz, J = 2.7 Hz ArH), 4.72 (s, 2H, CH_2_–CO), 3.84 (s, 3H, OCH_3_), 3.05 (t, 4H, J = 7.2 Hz Morpholine H), 1.19 (t, 4H, J = 7.2 Hz Morpholine H), ^13^C NMR (DMSO-d_6_, 100 MHz, δ ppm): 150.1, 148.7, 136.8, 129.3, 117.1, 110.3 (Ar–C), 192.3, 166.1, 128.8 (thiazolidine-C), 70.1, 70.1, 45.3, 45.3 (Morpholine-C), 163.9 (CO), 132.1 (CH), 57.2 (OCH_3_), 49.2 (CH_2_), Anal. Calcd. For C_17_H_18_N_2_O_5_S_2_: C, 51.71; H, 4.59; N, 7.09; S, 16.22; Found: C, 51.69; H, 4.61; N, 7.10; S,16.20.

### 2-Hydroxy-4-({[(Z)-5-(4-hydroxy-3-methoxybenzylidene)-4-oxo-2-thioxo-1,3-thiazolidin-3-yl]acetyl}amino)benzoic acid (3c)

Yield 59.2%, yellow solid, m.p 199–201℃, R_f_ 0.48 (ethyl acetate:pet-ether 2:1), IR (KBR) cm^−1^, 1610 (C=O), 1300 (C=S),1272 (C–N), 3130 (OH), 1370 (CH_3_), 1720 (COOH) ^1^H NMR (DMSO-d_6_, 400 MHz δ ppm) 12.0 (s, 1H, COOH), 9.75 (s, 1H, OH), 9.35 (d, 1H, OH), 7.75 (s, 1H, vinylic H), 7.41 (m, 1H, ArH), 7.19 (m, 1H, ArH), 7.05 (d,d 1H, J = 8.4 Hz, J = 2.7 Hz), 7.29–7.48 (m, 4H, ArH), 5.52 (s, 2H, CH_2_–CO), 3.82 (s, 3H, OCH_3_), ^13^C NMR (DMSO-d_6_, 100 MHz, δ ppm): 150.2, 149.7, 136.2, 131.3, 118.2, 109.5, 167.1, 141.4, 133.7, 119.7, 101.2, 100.1 (Ar–C), 197.2, 171.6, 130.3 (thiazolidine-C), 174.6 (COOH) 166.2 (CONH), 133.1 (CH), 57.0 (OCH_3_), 48.3 (CH_2_), Anal. Calcd. For C_20_H_16_N_2_O_7_S_2_: C, 52.12; H, 3.50; N, 6.08; S, 13.98; Found: C, 52.00; H, 3.55; N, 6.11; S, 14.01.

### 4-({[(Z)-5-(4-hydroxy-3-methoxybenzylidene)-4-oxo-2-thioxo-1,3-thiazolidin-3-yl]acetyl}amino)benzoic acid (3d)

Yield 84.5%, orange solid, m.p 217–219 ℃, R_f_ 0.45(ethyl acetate:pet-ether 2:1), IR (KBR) cm^−1^, 1622 (C=O), 1299 (C=S), 1050 (C–N), 3330 (OH), 1368 (CH_3_), 1704 (COOH) ^1^H NMR (DMSO-d_6_, 400 MHzδ ppm), 12.0 (s, 1H, CO–OH), 9.75 (s, 1H, OH) 7.71 (s, 1H, vinylic H), 7.41 (m, 1H, ArH), 7.19 (m, 1H, ArH), 7.05 (d,d 1H, J = 8.4 Hz, J = 2.7 Hz, ArH), 7.29–7.48 (m, 5H, ArH), 4.75 (s, 2H, CH_2_–CO), 3.83 (s, 3H, OCH_3_), ^13^C NMR (DMSO-d_6_, 100 MHz, δ ppm): 148.5, 147.3, 133.2, 128.6, 115.3, 108.8, 137.9, 130.4, 130.4, 123.2, 119.7, 119.7 (Ar–C), 193.3, 166.5, 129.4 (thiazolidine-C), 168.8 (COOH) 163.5 (CONH), 132.7 (CH), 56.4 (OCH_3_), 49.6 (CH_2_), Anal. Calcd. For C_20_H_16_N_2_O_6_S_2_: C, 53.99; H, 3.62; N, 6.29; S, 14.39; Found: C, 54.02; H, 3.60; N, 6.31; S, 14.33.

### (Z) 5-(4-methoxybenzylidene)-3-(2-anilino-2-oxoethyl)-2-thioxothiazolidin-4-one (3e)

Yield 60%, yellow solid m.p 190–192 °C, R_f_ 0.44 (ethyl acetate:pet-ether 2:1), IR (KBR) cm^−1^, 1620 (C=O), 1216 (C=S), 3368 (NH), 1022 (C–N), 1375 (CH_3_), ^1^H NMR (DMSO-d_6_, 400 MHzδ ppm) 7.56 (s, 1H, vinylic H) 7.29–7.48 (m, 5H, ArH), 7.10 (d, 2H, J = 8.7 Hz, ArH), 6.85 (d, 2H, J = 8.7 Hz, ArH), 5.63 (s, 2H, CH_2_–CO) and 3.71 (s, 3H, OCH_3_), ^13^C NMR (DMSO-d_6_, 100 MHz, δ ppm): 160.0, 132.3, 132.3, 125.8, 114.5, 114.5, 137.6, 129.2, 129.2, 124.6, 120.9, 120.9 (Ar–C), 194.2, 167.7, 129.8 (thiazolidine-C), 164.2 (CONH), 133.3 (CH), 55.7 (OCH_3_), 50.0 (CH_2_), Anal. Calcd. For C_19_H_16_N_2_O_3_S_2_: C, 59.30; H, 4.19; N, 7.28; S, 16.64; Found: C, 59.28; H, 4.20; N, 7.29; S, 16.61.

### (Z) 5-(4-methoxybenzylidene)-3-(2-morpholino-2-oxoethyl)-2-thioxothiazolidin-4-one (3f)

Yield 49.3%, yellow solid m.p 203–205 ℃, R_f_ 0.48 (ethyl acetate:pet-ether 2:1), IR (KBR) cm^−1^, 1650 (C=O), 1225 (C=S), 1101 (C–N), 1173 (C–O), 1375 (CH_3_), ^1^H NMR (DMSO-d_6_, 400 MHzδ ppm) 7.85 (s, 1H, vinylic H), 7.64 (d, 2H, J = 8.7 Hz ArH), 7.13 (d, 2H, J = 8.7 Hz ArH), 4.73 (s, 2H, CH_2_-CO), 3.84 (s, 3H, OCH_3_), 3.79 (t, 4H, J = 7.5 Hz Morpholine H), 1.19 (t, 4H, J = 7.5 Hz Morpholine H), ^13^C NMR (DMSO-d_6_, 100 MHz, δ ppm): 161.7, 133.3, 133.3, 127.2, 117.4, 117.4 (Ar–C), 190.8, 168.0, 128.3 (thiazolidine-C), 66.7, 66.7, 44.3 44.3 (Morpholine-C) 166.3 (CO), 134.9 (CH), 57.6 (OCH_3_), 45.2 (CH_2_), Anal. Calcd. For C_17_H_18_N_2_O_4_S_2_: C, 53.90; H, 4.79; N, 7.39; S, 16.91; Found: C, 53.87; H, 4.81; N, 7.37; S, 16.88.

### (Z) 5-(4-hydroxy-3-methoxybenzylidene)-3-(2-pyrrolidino-2-oxoethyl)-2-thioxothiazolidin-4-one (3g)

Yield 39.2%, yellow solid, m.p 187–189 ℃, R_f_ 0.31 (ethyl acetate:pet-ether 2:1), IR (KBR) cm^−1^, 1640 (C=O), 1215 (C=S),1260 (C–N), 3201 (OH), 1451 (CH_3_), ^1^H NMR (DMSO-d_6_, 400 MHzδ ppm) 9.76 (s, 1H, OH), 7.79 (s, 1H, vinylic H), 7.40 (m, 1H, ArH), 7.17 (m, 1H, ArH), 7.00 (d,d 1H, J = 8.4 Hz, J = 2.7 Hz, ArH), 4.72 (s, 2H, CH_2_–CO), 3.83 (d, 3H, OCH_3_), 3.04 (m, 4H, Pyrrolidine H),1.19 (t, 4H, J = 7.5 Hz, Pyrrolidine H), ^13^C NMR (DMSO-d_6_, 100 MHz, δ ppm): 159.8, 133.7, 133.7, 124.2, 115.1, 115.1 (Ar–C), 192.7, 165.3, 127.5 (thiazolidine-C), 48.5, 48.5, 23.7, 23.7 (pyrrolidine-C) 169.0 (CO), 134.7 (CH), 58.2 (OCH_3_), 51.3 (CH_2_), Anal. Calcd. For C_17_H_18_N_2_O_3_S_2_: C, 56.28; H, 5.00; N, 7.72; S, 17.65; Found: C, 56.31; H, 4.99; N, 7.70; S, 17.67.

### Enzyme inhibition studies

All required chemicals used in the enzyme extraction procedure were of high analytical grade. Enzyme inhibitory assay was performed on ELISA microplate reader at 340 nm and 96 well-plates were used for the sample analysis. Micropipettes from Gilson were used for sample loading. Sodium-d-glucoronate and d,l-glyceraldehyde were used as substrates along with a cofactor i.e. NADPH (nicotinamide adenine dinucleotide phosphate) from Sigma Aldrich.

### Extraction and purification of Aldehyde reductase (ALR1)

Aldehyde reductase enzyme was extracted from lamb kidney and the cortical part was separated carefully. The cortex was homogenized in triple volume of extraction buffer (2.0 mM EDTA, 0.25 M sucrose, 10 mM sodium phosphate and 2.5 mM β-mercaptoethanol at pH 7.2). The homogenate was centrifuged at 12,000 rpm at 4 °C for 30 min, the insoluble precipitates were discarded and the supernatant was saturated with 40%, 50% and 75% ammonium sulfate respectively and after each addition the solution was centrifuged at 12,000 rpm at 4 °C for 30 min, each time the pallet was discarded and for the last saturation the supernatant was taken and dialyzed overnight in extraction buffer. Next day the protein content was calculated via Bradford method and the crude aldehyde reductase was stored at − 80 °C [30].

### Extraction and purification of aldose reductase (ALR2)

The enzyme aldose reductase was extracted from calf lenses. 200–300 g lenses were added to triple volume of cold water and homogenized for 20 min. The homogenate was then centrifuged at 10,000 rpm for 15 min at 4 °C. The insoluble precipitates were discarded and the supernatant was saturated with 70% ammonium sulfate and after centrifugation at 10,000 rpm at 4 °C for 15 min the supernatant was dialyzed overnight and the protein contents was calculated via Bradford method and the crude aldose reductase was stored at −80 °C [31].

### ALR1 Enzyme inhibition assay

The assay was performed on ELISA (Bio-Tek ELx800TM Instrument, Inc. USA) based spectrophotometric analysis in 96 well plate. The assay mixture was composed of 20 µL buffer (100 mM potassium dihydrogen phosphate pH 6.2), 10 µL test compound (1 mM), 70 µL enzyme and incubated for 10 min at 37 °C followed by addition of 40 µL Glucoronate 50 mM (as a substrate) and 50 µL (0.5 mM) NADPH (nicotinamide adenine dinucleotide phosphate) as a co-factor. After 30 min incubation optical density was measured at 340 nm. Valproic acid was used as a positive control for ALR1 [32].

### ALR2 enzyme inhibition assay

The assay was performed on ELISA (Bio-Tek ELx800™ Instrument, Inc. USA) based spectrophotometric analysis in 96 well plate. The assay mixture was composed of 20 µL buffer (100 mM potassium dihydrogen phosphate pH 6.2), 10 µL test compound (1 mM), 70 µL enzyme and incubated for 10 min at 37 °C followed by addition of 40 µL substrate (d,l-glyceraldehyde 50 mM for ALR2) and 50 µL NADPH (0.5 mM) (nicotinamide adenine dinucleotide phosphate) as a co-factor. After 30 min incubation optical density was measured at 340 nm. Sulindac was used as positive control for ALR2 [33].

Results were analyzed by graph pad prism® software to calculate IC_50_ and percentage inhibition was calculated by the following formula.

% Inhibition = [100−(Absorbance test well/Absorbance control)] × 100.

### Molecular docking studies

For docking studies, crystal structures of human aldose reductase (PDB ID: **1US0**) [33] and aldehyde reductase (PDB ID: **3FX4**) [25] were used. Structures of the tested compounds were drawn by MOE builder tool [34] and optimization was achieved using MMFF94x forcefield [35]. Afterwards the energy minimization of the target proteins was carried out by Molecular Operating Environment [36]. LeadIT (BioSolveIT GmbH, Germany) [37] was used to perform docking analysis of the prepared ligands inside the respective receptors. Load Receptor Utility of the LeadIT software was used to load the receptors. Active pocket of the proteins for docking analysis was identified by keeping the amino acid residues in 10.0 Å radius and keeping co-factor (NADPH) within ALR1 and ALR2. Values of the amino acid flips and water handling were kept as by default. Once docking analysis was completed, the possible interactions of ligands with receptor proteins were inspected for studying the possible interactions using HYDE assessment [37]. Discovery Studio Visualizer was used to perform visualize the interactions of ligand and receptors [38].

### Molecular dynamics simulations

All MD simulations were carried out using the PMEMD (Particle Mesh Ewald Molecular Dynamics) module of AMBER 18 simulation package [39]. The crystallographic structures of ALR1 (3FX4) and ALR2 (1US0) were downloaded from RCSB protein databank, the protein structures were protonated by using the H++ webserver [40]. Each complex system was solvated in a cubic box of water molecules. The ff14SB4 [41] force filed was used for proteins while for ligands the second-generation of the General Amber Force Filed (GAFF2) [42] with AM1-BCC charges and TIP3P [43] for water molecules. The GAFF parameters and coordinates were generated by using the antechamber [44] and xleap modules of AMBER 18 simulation package. To neutralize the total charge of each system only sodium as a counter ion was added with parameters from Joung and Cheatham [45]. For post-simulation inspection, VMD was used [46].

For equilibration each system was minimized with 500 steps of steepest decent minimization with 2.0 kcal mol^−1^ restrains on the heavy atoms of protein. Then the systems were heated for 100 ps from 10 to 300 K temperature using 2.0 kcal mol^−1^ as a restraint on protein in NVT ensemble with Langevin thermostat [47] and collision frequency is 5 ps^−1^. After that 20 ns MD simulation was carried out using Berendsen barostat [48] and time constant was 2 ps in NPT ensemble. Moreover 100 ps equilibration was done in NPT ensemble without any restrain. For data collection 20 ns MD simulation have been carried out in NPT ensemble without restrain with time step of 2 fs. The temperature and pressure during these MD simulations is 300 K and 1 bar respectively. Furthermore, SHAKE protocol was used involving all hydrogen bonded atoms, 10 Å non-bonded cut-off and particle mesh Ewald (PME) methodology [49] to calculate the long-range electrostatic interactions with periodic boundary condition was used. Finally, the trajectories of these MD simulations were analyzed using CPPTRAJ [50] module of AMBER.

#### ADME properties

ADME (Absorption, Distribution, Metabolism, and Excretion) properties of the newly synthesized molecules were predicted using previous protocols and predictions tools [26, 51–54].

## Supplementary Information

**Additional file 1:** **Figure S1.** 2D interactions of cognate ligand (**FX4401**) (**a**), selective inhibitor (**3e**) of ALR1 (**b**) and dual inhibitors (**c**: **3f**; **d**; **3g**) inside the active pocket of 3FX4.

## Data Availability

All the relevant data supporting the conclusions of this article is included in the article.

## Abbreviations

ALR1: Aldehyde reductase
ALR2: Aldose reductase
ARIs: AR inhibitors
NADPH: Nicotinamide adenine dinucleotide phosphate
JNK: C-Jun N-terminal kinase
JSP-1: JNK-stimulating phosphatase-1
cAMP: Cyclic adenosine monophosphate
FTIR: Fourier transform infrared
^1^H NMR: Proton nuclear magnetic resonance
Rf: Relative factor
mol.: Moles
RMSD: Root mean square deviation
RMSF: Root mean square fluctuation

## References

[1] Sarwar N, Gao P, Seshasai SR, Gobin R, Kaptoge S, Di Angelantonio E, Ingelsson E, Lawlor DA, Selvin E, Stampfer M, Stehouwer CD, Lewington S, Pennells L, Thompson A, Sattar N, White IR, Ray KK, Danesh J (2010). Diabetes mellitus, fasting blood glucose concentration, and risk of vascular disease: a collaborative meta-analysis of 102 prospective studies. Lancet.

[2] Mergenthaler P, Lindauer U, Dienel GA, Meisel A (2013). Sugar for the brain: the role of glucose in physiological and pathological brain function. Trends Neurosci.

[3] Niedowicz DM, Daleke DL (2005). The role of oxidative stress in diabetic complications. Cell Biochem Biophys.

[4] Yabe-Nishimura C (1998). Aldose reductase in glucose toxicity: a potential target for the prevention of diabetic complications. Pharmacol Rev.

[5] Brownlee M (2001). Biochemistry and molecular cell biology of diabetic complications. Nature.

[6] Wiernsperger NF (2003). Oxidative stress as a therapeutic target in diabetes: revisiting the controversy. Diabetes Metab.

[7] Purves T, Middlemas A, Agthong S, Jude EB, Boulton AJ, Fernyhough P, Tomlinson DR (2001). A role for mitogen-activated protein kinases in the etiology of diabetic neuropathy. FASEB J.

[8] Williamson JR, Chang K, Frangos M, Hasan KS, Ido Y, Kawamura T, Nyengaard JR, van Den Enden M, Kilo C, Tilton RG (1993). Hyperglycemic pseudohypoxia and diabetic complications. Diabetes.

[9] Tang WH, Martin KA, Hwa J (2012). Aldose reductase, oxidative stress, and diabetic mellitus. Front Pharmacol.

[10] Obrosova IG (2005). Increased sorbitol pathway activity generates oxidative stress in tissue sites for diabetic complications. Antioxid Redox Signal.

[11] Bruno G, Costantino L, Curinga C, Maccari R, Monforte F, Nicolo F, Ottana R, Vigorita M (2002). Synthesis and aldose reductase inhibitory activity of 5-arylidene-2, 4-thiazolidinediones. Bioorg Med Chem.

[12] Tuomi T, Santoro N, Caprio S, Cai M, Weng J, Groop L (2014). The many faces of diabetes: a disease with increasing heterogeneity. Lancet.

[13] Lee I-M, Shiroma EJ, Lobelo F, Puska P, Blair SN, Katzmarzyk PT, Group LPASW (2012). Effect of physical inactivity on major non-communicable diseases worldwide: an analysis of burden of disease and life expectancy. The Lancet.

[14] Del-Corso A, Balestri F, Di Bugno E, Moschini R, Cappiello M, Sartini S, La-Motta C, Da-Settimo F, Mura U (2013). A new approach to control the enigmatic activity of aldose reductase. PLoS ONE.

[15] Smith TK, Young BL, Denton H, Hughes DL, Wagner GK (2009). First small molecular inhibitors of *T. brucei* dolicholphosphate mannose synthase (DPMS), a validated drug target in African sleeping sickness. Bioorg Med Chem Lett.

[16] Dolezel J, Hirsova P, Opletalova V, Dohnal J, Marcela V, Kunes J, Jampilek J (2009). Rhodanineacetic acid derivatives as potential drugs: preparation, hydrophobic properties and antifungal activity of (5-arylalkylidene-4-oxo-2-thioxo-1, 3-thiazolidin-3-yl) acetic acids. Molecules.

[17] Zervosen A, Lu W-P, Chen Z, White RE, Demuth TP, Frère J-M (2004). Interactions between penicillin-binding proteins (PBPs) and two novel classes of PBP inhibitors, arylalkylidene rhodanines and arylalkylidene iminothiazolidin-4-ones. Antimicrob Agents Chemother.

[18] Cutshall NS, O’Day C, Prezhdo M (2005). Rhodanine derivatives as inhibitors of JSP-1. Bioorg Med Chem Lett.

[19] Free CA, Majchrowicz E, Hess SM (1971). Mechanism of inhibition of histidine decarboxylase by rhodanines. Biochem Pharmacol.

[20] Wang L, Kong F, Kokoski CL, Andrews DW, Xing C (2008). Development of dimeric modulators for anti-apoptotic Bcl-2 proteins. Bioorg Med Chem Lett.

[21] Tomašić T, Zidar N, Mueller-Premru M, Kikelj D, Mašič LP (2010). Synthesis and antibacterial activity of 5-ylidenethiazolidin-4-ones and 5-benzylidene-4,6-pyrimidinediones. Eur J Med Chem.

[22] Sortino M, Delgado P, Juárez S, Quiroga J, Abonía R, Insuasty B, Nogueras M, Rodero L, Garibotto FM, Enriz RD (2007). Synthesis and antifungal activity of (Z)-5-arylidenerhodanines. Bioorg Med Chem.

[23] Rajamaki S, Innitzer A, Falciani C, Tintori C, Christ F, Witvrouw M, Debyser Z, Massa S, Botta M (2009). Exploration of novel thiobarbituric acid-, rhodanine-and thiohydantoin-based HIV-1 integrase inhibitors. Bioorg Med Chem Lett.

[24] Carbone V, Zhao H-T, Chung R, Endo S, Hara A, El-Kabbani O (2009). Correlation of binding constants and molecular modelling of inhibitors in the active sites of aldose reductase and aldehyde reductase. Bioorg Med Chem.

[25] Carbone V, Giglio M, Chung R, Huyton T, Adams J, Maccari R, Ottana R, Hara A, El-Kabbani O (2010). Structure of aldehyde reductase in ternary complex with a 5-arylidene-2, 4-thiazolidinedione aldose reductase inhibitor. Eur J Med Chem.

[26] Daina A, Michielin O, Zoete V (2017). Swiss ADME: a free web tool to evaluate pharmacokinetics, drug-likeness and medicinal chemistry friendliness of small molecules. Sci Rep.

[27] Wang S, Zhao Y, Zhu W, Liu Y, Guo K, Gong P (2012). Synthesis and anticancer activity of Indolin-2-one derivatives bearing the 4-thiazolidinone moiety. Arch Pharm.

[28] Toubal K, Djafri A, Chouaih A, Talbi A (2012). Synthesis and structural determination of novel 5-arylidene-3-N (2-alkyloxyaryl)-2-thioxothiazolidin-4-ones. Molecules.

[29] Montalbetti CA, Falque V (2005). Amide bond formation and peptide coupling. Tetrahedron.

[30] Ward WH, Sennitt CM, Ross H, Dingle A, Timms D, Mirrlees DJ, Tuffin DP (1990). Ponalrestat: a potent and specific inhibitor of aldose reductase. Biochem Pharmacol.

[31] Kador PF, Kinoshita J, Brittain D, Mirrlees D, Sennitt C, Stribling D (1986). Purified rat lens aldose reductase. Polyol production in vitro and its inhibition by aldose reductase inhibitors. Biochem J.

[32] Hayman S, Kinoshita JH (1965). Isolation and properties of lens aldose reductase. J Biol.

[33] Saraswat M, Muthenna P, Suryanarayana P, Petrash JM, Reddy GB (2008). Dietary sources of aldose reductase inhibitors: prospects for alleviating diabetic complications. Asia Pac J Clin Nutr.

[34] Chemical Computing Group's Molecular Operating Environment (MOE) MOE 2019. 0201. http://www.chemcomp.com/MOEMolecular_Operating_Environment.htm. Accessed 11 Jan 2020

[35] Labute P (2007) Protonate 3D, Chemical Computing Group. http://www.chemcomp.com/journal/proton.htm. Accessed 11 Jan 2020

[36] Schneider N, Lange G, Hindle S, Klein R, Rarey M (2013). A consistent description of HYdrogen bond and DEhydration energies in protein–ligand complexes: methods behind the HYDE scoring function. J Comput Aided Mol Des.

[37] LeadIT version 2.3.2; BioSolveIT GmbH, Sankt Augustin, Germany, 2017, www.biosolveit.de/LeadIT

[38] BIOVIA Discovery Studio Client v19.1.0.18287. Accelrys Discovery Studio. Accelrys Software Inc, San Diego, 2019.

[39] Case DA, Ben-Shalom IY, Brozell SR, Cerutti DS, Cheatham TE, Cruzeiro VWD, Darden TA, Duke RE, Ghoreishi D, Gilson MK, Gohlke H, Goetz AW, Greene D, Harris R, Homeyer N, Huang Y, Izadi S, Kovalenko A, Kurtzman T, Lee TS, LeGrand S, Li P, Lin C, Liu J, Luchko T, Luo R, Mermelstein DJ, Merz KM, Miao Y, Monard G, Nguyen C, Nguyen H, Omelyan I, Onufriev A, Pan F, Qi R, Roe DR, Roitberg A, Sagui C, Schott-Verdugo S, Shen J, Simmerling CL, Smith J, Salomon- Ferrer R, Swails J, Walker RC, Wang J, Wei H, Wolf RM, Wu X, Xiao L, York DM, Kollman PA (2018) AMBER 2018, University of California, San Francisco

[40] Gordon JC, Myers JB, Folta T, Shoja V, Heath LS, Onufriev A (2005). H++: a server for estimating pKas and adding missing hydrogens to macromolecules. Nucleic Acids Res.

[41] Maier JA, Martinez C, Kasavajhala K, Wickstrom L, Hauser KE, Simmerling C (2015). ff14SB: improving the accuracy of protein side chain and backbone parameters from ff99SB. J Chem Theory Comput.

[42] Träg J, Zahn D (2019). Improved GAFF2 parameters for fluorinated alkanes and mixed hydro- and fluorocarbons. J Mol Model.

[43] Jorgensen WL, Chandrasekhar J, Madura JD, Impey RW, Klein ML (1983). Comparison of simple potential functions for simulating liquid water. J Chem Phys.

[44] Wang J, Wang W, Kollman PA, Case DA (2006). Automatic atom type and bond type perception in molecular mechanical calculations. J Mol Graph Model.

[45] Joung IS, Cheatham TE (2008). Determination of alkali and halide monovalent ion parameters for use in explicitly solvated biomolecular simulations. J Phys Chem B.

[46] Humphrey W, Dalke A, Schulten K (1996). VMD: visual molecular dynamics. J Mol Graph.

[47] Loncharich RJ, Brooks BR, Pastor RW (1992). Langevin dynamics of peptides: the frictional dependence of isomerization rates of N-acetylalanyl-Nʹ-methylamide. Biopolymers.

[48] Lin Y, Pan D, Li J, Zhang L, Shao X (2017). Application of Berendsen barostat in dissipative particle dynamics for nonequilibrium dynamic simulation. J Chem Phys.

[49] Darden T, York D, Pedersen L (1993). Particle mesh Ewald: an N⋅log(N) method for Ewald sums in large systems. J Chem Phys.

[50] Roe DR, Cheatham TE (2013). PTRAJ and CPPTRAJ: Software for processing and analysis of molecular dynamics trajectory data. J Chem Theory Comput.

[51] Daina A, Michielin O, Zoete V (2014). iLOGP: a simple, robust, and efficient description of n-octanol/water partition coefficient for drug design using the GB/SA approach. J Chem Inf Model.

[52] Daina A, Zoete V (2016). A BOILED-Egg to predict gastrointestinal absorption and brain penetration of small molecules. Chem Med Chem.

[53] Ertl P, Rohde B, Selzer BP (2000). Fast calculation of molecular polar surface area as a sum of fragment-based contributions and its application to the prediction of drug transport properties. J Med Chem.

[54] Khan I, Khan A, Halim SA, Khan M, Zaib S, Essa B, Al-Yahyaei M, Al-Harrasi A, Ibrar A (2021). Utilization of the common functional groups in bioactive molecules: Exploring dual inhibitory potential and computational analysis of keto esters against α-glucosidase and carbonic anhydrase-II enzymes. Int J Biol Macromol.

