# Supplementary material for: Rhodanine-3-acetamide derivatives as aldose and aldehyde reductase inhibitors to treat diabetic complications: synthesis, biological evaluation, molecular docking and simulation studies
Source: BMC Chem. 2021 Apr 27;15(1):28. doi: 10.1186/s13065-021-00756-z (PMC8080350; doi:10.1186/s13065-021-00756-z)
Supplement: Supplementary file 1 — Additional file 1: Figure S1. 2D interactions of cognate ligand (FX4401) (a), selective inhibitor (3e) of ALR1 (b) and dual inhibitors (c: 3f; d; 3g) inside the active pocket of 3FX4. [file 13065_2021_756_MOESM1_ESM.docx]

**Supporting information**

| 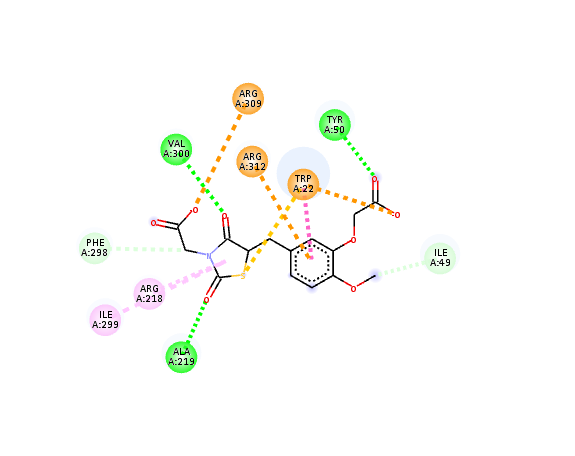  (a) | 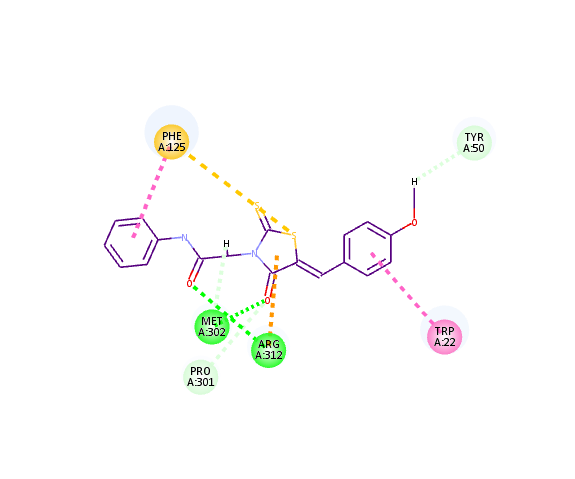  (b) |
| --- | --- |
| 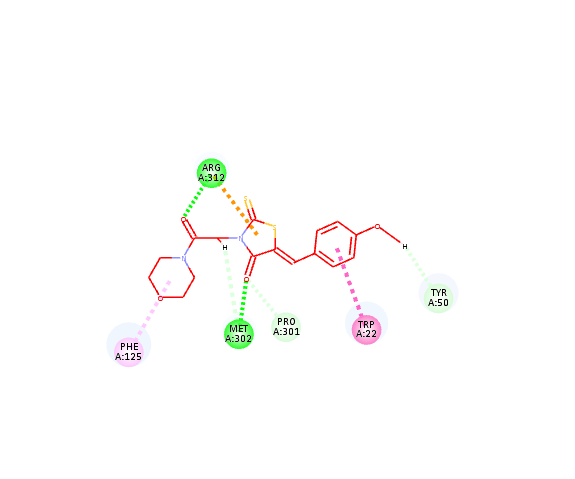  (c) | 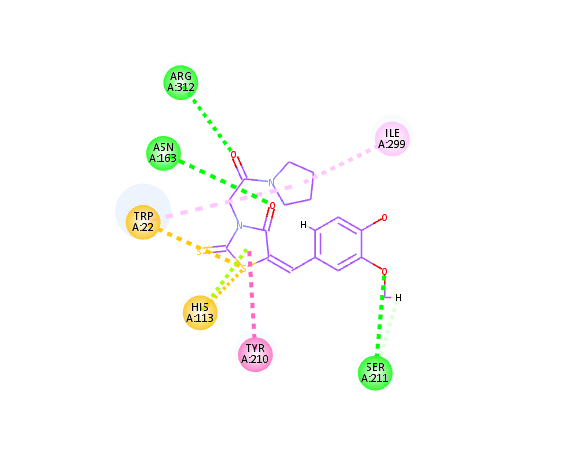  (d) |
| **Figure S1.** 2D interactions of cognate ligand (**FX4401**) (a), selective inhibitor (**3e**) of ALR1 (b) and dual inhibitors (c: **3f**; d; **3g**) inside the active pocket of 3FX4. | |

| 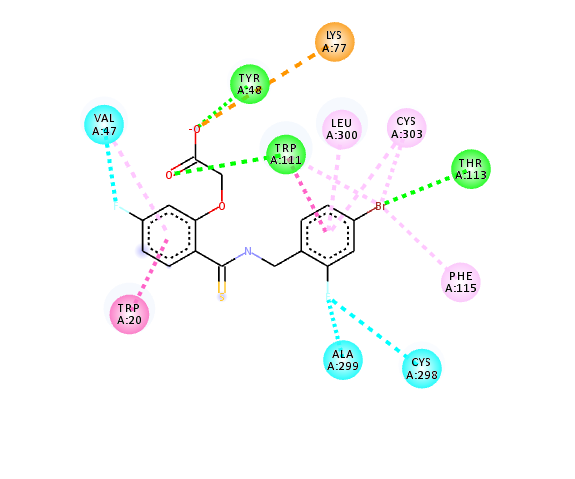  (a) | 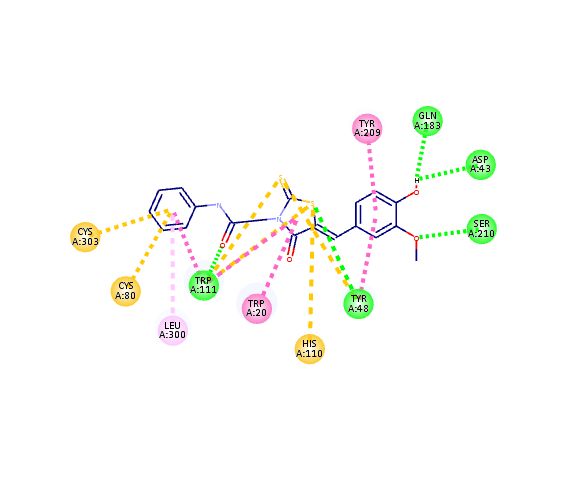  (b) |
| --- | --- |
| 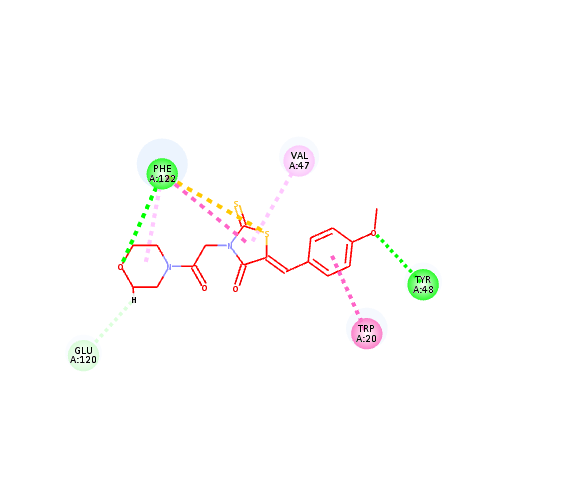  (c) | 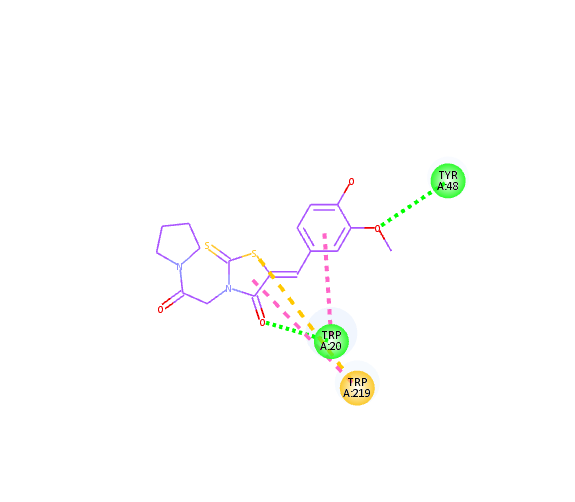  (d) |
| **Figure S2.** 2D interactions of cognate ligand (IDD594) (a), selective inhibitor (**3a**) of ALR2 (b) and dual inhibitors (c: **3f**; d; **3g**) inside the active pocket of 1US0. | |
